# Turbulence-Resistant Femtosecond Filaments via Nonlinear Self-Guiding and OAM Modulation

**DOI:** 10.3390/s26092618

**Published:** 2026-04-23

**Authors:** Jinpei Liu, Xi Yang, Weiyun Jin, Zuyou Ren, Caiyi Yang, Tingting Shi

**Affiliations:** 1 College of Information Engineering, Hebei GEO University, Shijiazhuang 052161, China; 002924@hgu.edu.cn (W.J.); 423109110116@hgu.edu.cn (Z.R.); 423109110305@hgu.edu.cn (C.Y.); 423109110219@hgu.edu.cn (T.S.); 2 Intelligent Sensor Network Engineering Research Center of Hebei Province, Shijiazhuang 052161, China

**Keywords:** femtosecond laser filamentation, turbulence, nonlinear self-guiding effect, orbital angular momentum, multiple filaments

## Abstract

As a prominent frontier in ultrafast laser–matter interaction, femtosecond laser filamentation holds great potential for atmospheric pollutant detection and remote sensing. However, its practical application in the open atmosphere is severely hampered by atmospheric turbulence, which induces beam wander, wavefront distortion, and intensity scintillations. In this study, we numerically investigated the propagation dynamics of femtosecond laser filaments in a turbulent medium and elucidated the underlying physical mechanisms. The results show that, compared to linear propagation, the nonlinear self-guiding effect inherent to filamentation effectively suppresses turbulence-induced beam wander. Furthermore, by employing vortex beams carrying orbital angular momentum (OAM), we significantly suppressed the stochastic generation of multiple filaments, thereby notably improving the stability of long-range filament propagation in complex atmospheric conditions. These findings provide new insights into the physical mechanisms and novel strategies for improving the robustness of laser filamentation technology in real-world turbulent environments.

## 1. Introduction

As a frontier topic in ultrafast laser–matter interaction, femtosecond laser filamentation exhibits considerable potential for environmental monitoring. Achieving long-range, highly efficient, and high-intensity propagation of laser energy in free space remains a fundamental challenge in various cutting-edge fields, including pollutant detection and atmospheric remote sensing. Traditional high-power laser beams inevitably suffer from diffraction-induced beam divergence during long-range propagation, thereby resulting in a rapid decrease in intensity and severely limiting the effective interaction distance. To overcome this physical bottleneck, femtosecond laser filamentation has emerged as a promising solution, garnering extensive attention from researchers [1,2,3]. For a Gaussian beam, the critical power is given by $P_{cr}=3.77\lambda^{2}/\left(8\pi n_{0}n_{2}\right)$ [4], where $P_{cr}$ is the critical power for self-focusing, λ is the laser wavelength, $n_{0}$ is the linear refractive index of air, and $n_{2}$ is the nonlinear refractive index coefficient), and the laser can propagate as filaments [5,6]. When the beam power reaches the critical power for self-focusing, the laser can propagate as filaments, maintaining a high power density (approximately $10^{13}$ W/cm^2^) over distances far exceeding the Rayleigh length. This phenomenon primarily arises from a dynamic balance between Kerr self-focusing, plasma defocusing, and the higher-order Kerr effect (HOKE) induced by the intense laser field [7,8]. Leveraging its unique capability for remote high-intensity delivery, femtosecond laser filamentation provides a revolutionary approach to pollutant detection [9,10,11] and atmospheric remote sensing [12,13,14,15,16].

However, atmospheric turbulence continues to be a pivotal factor affecting the performance of femtosecond laser filamentation. Turbulent fluctuations in pressure, temperature, and other physical parameters induce random fluctuations in the refractive index, effectively transforming the atmosphere into a stochastic optical medium with a temporally and spatially varying refractive index. This phenomenon significantly compromises beam propagation stability and, consequently, the performance of femtosecond laser applications over long distances. Existing experimental and theoretical studies have investigated the impact of atmospheric turbulence on filament length [17,18], the transverse drift of the filament centroid [19,20], as well as the generation of multiple filaments and the enhancement of filament-induced fluorescence [21,22]. Minor perturbations in the filamentation process can profoundly degrade the sensitivity of remote sensing applications. While previous research has demonstrated that turbulence induces transverse drift of the filament centroid, it remains unclear whether turbulence exerts a similar effect on beam propagation in linear and nonlinear regimes. Moreover, the effect of the spatial position of the turbulent layer on beam wander—a factor critical to the application of filament-based remote sensing of atmospheric pollutants—has not been fully elucidated.

More critically, atmospheric turbulence randomizes the originally ordered single-beam energy distribution. This perturbation can lead to multiple energy hot spots and induce uncontrollable multiple filamentation, which disperses the laser energy into several unstable propagation channels and thereby significantly compromises the effectiveness of remote detection [21,23]. To address these formidable challenges, researchers have increasingly turned their attention to non-traditional beams with specialized spatial structures. Among these, vortex beams carrying orbital angular momentum (OAM) have garnered considerable interest due to their unique physical characteristics. Unlike conventional Gaussian beams with a peaked intensity profile, vortex beams are characterized by a helical phase front, which is described by the phase factor exp(ilφ) (where *l* is the topological charge), and possess a central intensity null, known as a phase singularity [24]. This distinctive annular intensity profile, coupled with an inherent rotational phase structure, suggests that they exhibit fundamentally different propagation dynamics in nonlinear media compared to Gaussian beams. Preliminary studies have demonstrated that femtosecond vortex beams can also undergo self-focusing in air, forming unique ring-like or multiple-filament arrays [25,26]. These observations have led to a compelling scientific question: Could the unique phase and intensity distributions of vortex beams confer superior robustness against turbulence, potentially outperforming Gaussian beams in maintaining filament stability?

To fill the aforementioned research gaps, we aimed to investigate, through large-scale, high-precision numerical simulations, the long-range nonlinear propagation of femtosecond laser pulses in atmospheric turbulence of varying intensities and the filamentation dynamics of femtosecond vortex laser pulses in such turbulent environments. Under both linear and nonlinear propagation conditions, we simulated the effects of turbulence location and intensity on the transverse drift of the beam centroid. Our results show that the nonlinear effects inherent to femtosecond laser filaments can suppress beam modulation induced by atmospheric turbulence. Concurrently, this study simulated and explored the influence of atmospheric turbulence on the transverse drift of vortex beams during nonlinear propagation. In our simulations, we observed that introducing vortex beams carrying orbital angular momentum can effectively suppress the random generation of multiple filaments. The research demonstrates that vortex beams with topological charge can reduce the adverse effects induced by atmospheric turbulence during propagation, thereby enhancing the stability of filamentary transmission in the atmosphere. These studies contribute to improving the accuracy of Light Detection and Ranging (LIDAR) technology and hold significant importance for the fields of pollutant detection and atmospheric remote sensing.

## 2. The Theoretical Models

To investigate the filamentation dynamics in atmospheric turbulence, numerical simulations were performed using the (2 + 1)-dimensional nonlinear wave equation [27,28]:

(1) $$2ik_{0}\frac{\partial A}{\partial Z}+\left(\frac{\partial^{2}}{\partial x^{2}}+\frac{\partial^{2}}{\partial y^{2}}\right)A+2\frac{k_{0}^{2}}{n_{0}}\left[∆n+\tilde{n}(x,y,z_{0})\right]A=0,$$

*k*_0_ is the wavenumber, *A* is the optical field amplitude, *n*_0_ is the refractive index of air, *n* denotes the refractive index fluctuation in air induced by atmospheric turbulence, and *z*_0_ is the effective propagation distance. The initial light field amplitude for the Gaussian beam is given by $A_{G}(r,0)=\sqrt{I_{0}}exp(-r^{2}/w_{0}^{2})$ and for the vortex beam with topological charge l, it is given by $A_{V}(r,\varphi ,0)=\sqrt{I_{0}}exp(-r^{2}/w_{0}^{2})exp(il\varphi )$. In the simulations, the central wavelength is λ=800 nm, and the beam waist radius is set to 1 mm. When the optical power is sufficiently high, the change in the refractive index Δn arising from nonlinear effects must be considered. This Δn includes the nonlinear refractive index change corresponding to the Kerr self-focusing effect ($\Delta n_{kerr}=n_{2}I$, where $n_{2}=2\times 10^{-19}$ cm^2^/W) and the equivalent refractive index change induced by plasma defocusing ($∆n_{plasma}=-\gamma I^{8}$) [7]. Here, *γ* is an empirical parameter introduced to model the effective plasma defocusing driven by 8-photon ionization. Its value is calibrated to match the intensity clamping threshold observed in filamentation experiments. Specifically, *γ* is chosen such that the plasma-induced defocusing term counteracts the Kerr effect ($\Delta n_{kerr}=n_{2}I$) at high intensities, limiting the peak intensity to approximately $5\times 10^{13}$ W/cm^2^, which is consistent with the general intensity clamping levels observed in filamentation experiments [2]. The laser intensity I is proportional to the square of the field amplitude, $I\propto {\left|A\right|}^{2}$, within the framework of Equation (1). This definition is consistent with standard theoretical models for nonlinear propagation [29]. Note that the temporal effects of nonlinear propagation are not accounted for in Equation (1), as this study primarily focuses on the spatial dynamics of filaments influenced by atmospheric turbulence. The validity of this simplified model is confirmed in previous research [28,30]. Specifically, within the ultra-short duration of a femtosecond pulse, the atmospheric turbulence is effectively treated as ‘frozen’, and this quasi-static approximation is widely adopted in the simulation of femtosecond pulse propagation in turbulent media [22,31].

The modified von Kármán spectrum model was employed to simulate refractive index fluctuations induced by atmospheric turbulence. Turbulent phase screens, which serve as discrete propagation units, are used to model the energy cascade from the outer scale ($L_{0}=1$ m in our simulations) down to the inner scale ($l_{0}=1$ mm). The presence of turbulence modifies atmospheric pressure distribution and local temperature, thereby inducing refractive index variations. The beam propagation range was discretized according to the unit propagation distance (Δ*z*) of the turbulence phase screen. By utilizing various random number sequences, we generated random turbulence phase screens that represent the cumulative phase variations across the (*x*, *y*) plane over a propagation distance of 1 m. Based on the modified von Kármán spectrum model, the power spectral density of phase fluctuation on each random turbulence phase screen (in the (*x*, *y*) plane) can be expressed as follows:

(2) $$F_{\emptyset }\left(\kappa_{x},\kappa_{y}\right)=2\pi k_{0}^{2}\Delta z\phi_{n}(\kappa_{x},\kappa_{y},0),$$

$F_{\phi }(\kappa_{x},\kappa_{y})$ denotes the two-dimensional power spectral density of phase fluctuations on the phase screen. The term $\phi_{n}(\kappa_{x},\kappa_{y},0)$ is the three-dimensional power spectrum of refractive index fluctuations. Based on the modified von Kármán spectrum model, $\phi_{n}(\kappa )$ is expressed as $\phi_{n}(\kappa )=0.033C_{n}^{2}(\kappa^{2}+\kappa_{0}^{2}{)}^{-11/6}exp(-\kappa^{2}/\kappa_{m}^{2})$, where κ is the spatial wavenumber, $\kappa_{0}=2\pi /L_{0}$, $\kappa_{m}=5.92/l_{0}$, and $L_{0}$ and $l_{0}$ are the outer and inner scales of turbulence, respectively [32].

**Figure 1:**
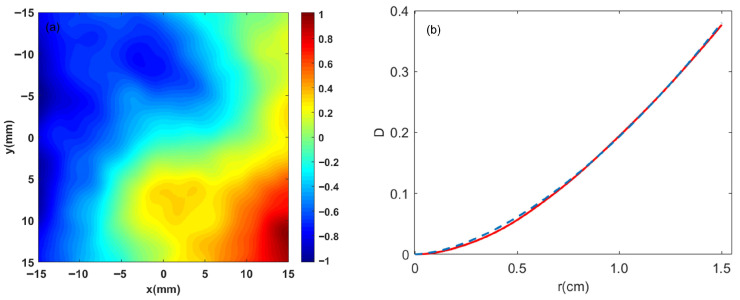
(**a**) Representative turbulent phase screen characterized by a refractive index structure constant ${C_{n}^{2}=1.0\times 10}^{-13}$ cm^−2/3^ at a propagation distance of z=1 m (phase expressed in radians). (**b**) Comparative analysis of the phase structure function: the solid red line denotes the numerical simulation results, while the blue dashed line represents the theoretical predictions.

Figure 1a shows a random phase screen of turbulence with a refractive index structure constant of $C_{n}^{2}={1.0\times 10}^{-13}$ cm^−2/3^ and a separation distance of Δ*z* = 1 m. In the simulation of laser propagation, such a random phase screen is inserted at intervals of 1 m along the beam path. Thus, two screens are utilized for a 2 m propagation distance. To investigate the statistical properties of turbulence-induced multiple filaments, numerical simulations were performed with 30 independent realizations of random phase screens for each level of atmospheric turbulence strength.

The validity of the phase screen generated by this method can be validated against the theoretical values of the phase structure function, which is given by [33]

(3) $$D\left(r\right)=\langle {\left[\varphi \left(r^{\prime }\right)-\varphi \left(r^{\prime }+r\right)\right]}^{2}\rangle ={6.88(r/r_{0})}^{5/3},$$

where the angle brackets <∙> denote the statistical average over all random turbulence phase screen realizations, $r_{0}$ represents the Fried parameter and *r* denotes the distance between any two points in the phase screen, and the constant 6.88 is the Kolmogorov constant for the phase structure function [34]. As shown in Figure 1b, the calculated phase structure function from the simulated turbulence phase screens is compared with the theoretical curve. The red solid line, derived from averaging the phase structure functions of 30 independent random phase screen realizations, represents the simulated results. The blue dashed line corresponds to the theoretical values calculated using Equation (3). The minor discrepancies between the solid and dashed lines in Figure 1b are attributed to the finite size of the phase screens, which is constrained by available computational resources.

## 3. Results and Discussion

### 3.1. Effect of Turbulence Location and Intensity on Beam Wander

The optical property variations induced by atmospheric turbulence, which manifest as random fluctuations in the refractive index, were simulated by generating random phase screens based on a modified von Karman spectrum model. To examine the effect of turbulence location, we varied the position of the phase screen along the laser propagation path to positions of 2, 4, 6, 8, and 10 m. The centroid wander of the laser spot was then analyzed at the focal plane of the optical system (10 m from the source). For each specified turbulence strength, we conducted 30 independent numerical simulations, each employing a distinct random phase screen, and then performed a statistical analysis of the results.

**Figure 2:**
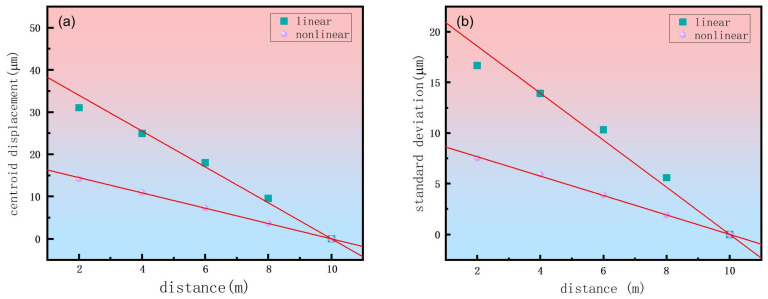
(**a**) The centroid displacement and (**b**) its standard deviation of a laser spot at the 10-m focal plane, induced by varying the axial position of turbulence, for a refractive index structure constant of 10^−12^ cm^−2/3^. The red solid lines represent linear fits to the simulated data.

Subsequently, along the 10-m propagation path, turbulence phase screens were placed at 2 m intervals. Results were statistically summarized from 30 realizations of random phase screens and we computed the standard deviation of the beam centroid displacement caused by turbulence at each location. A comparison between linear and nonlinear propagation regimes was then conducted. When the optical power is sufficiently high, the change in the refractive index Δn arising from nonlinear effects must be considered. This Δn includes the nonlinear refractive index change corresponding to the Kerr self-focusing effect and the equivalent refractive index change induced by plasma defocusing. To compare linear and nonlinear propagation regimes, the simulations are performed under two conditions: for linear propagation, the nonlinear term Δn is set to zero; for nonlinear propagation, the full nonlinear term Δn is included. As shown in Figure 2, we present separately, for both linear and nonlinear propagation, the beam centroid displacement and its standard deviation at the system’s focal plane (located 10 m away), each induced by turbulence at different phase-screen locations.

As shown in Figure 2, the linear fits to the numerically simulated beam centroid displacement and its standard deviation both show a negative correlation with the turbulence interaction position, with a confidence level exceeding 98%. This indicates that as the turbulence interaction position moves closer to the focal plane (at 10 m) of the optical system, the turbulence-induced beam centroid displacement and its standard deviation decrease. At the 10 m position, the turbulence-induced beam centroid displacement and its standard deviation become comparable to those resulting from the inherent laboratory turbulence without externally applied turbulence. The closer the turbulence-induced spatial modulation of the optical field is to the initial field, the more significant its impact on femtosecond laser filamentation. This occurs because modulation near the initial field allows the Kerr effect to induce energy competition between each ‘hot spot’ and the surrounding background reservoir. Consequently, a dynamic equilibrium is established in which no single ‘hot spot’ dominates, thereby suppressing filament formation and enhancing beam wander. Conversely, beam wander decreases as the turbulence interaction region approaches the focal plane. Therefore, beam wander during propagation depends on the turbulence interaction position.

**Figure 3:**
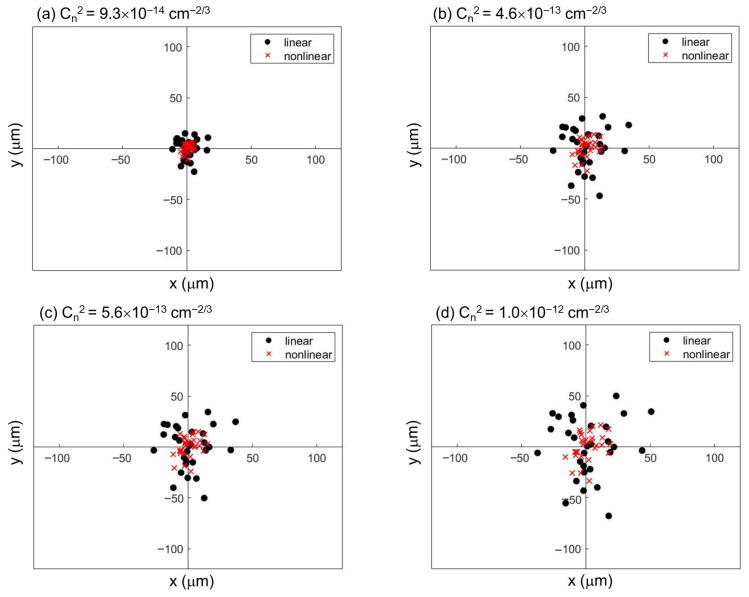
Two-dimensional spatial coordinates of the beam centroid at a propagation distance of 10 m for different values of the refractive index structure constant, $C_{n}^{2}$ (in units of cm^−2/3^). The black and red dots denote the centroid positions under linear and nonlinear propagation regimes, respectively. (**a**) ${C_{n}^{2}=9.3\times 10}^{-14}$ cm^−2/3^, (**b**) ${C_{n}^{2}=4.6\times 10}^{-13}$ cm^−2/3^, (**c**) ${C_{n}^{2}=5.6\times 10}^{-13}$ cm^−2/3^, (**d**) ${C_{n}^{2}=1.0\times 10}^{-12}$ cm^−2/3^.

A comparison of the beam centroid displacement and its standard deviation under identical turbulence conditions (Figure 2) reveals that the formation of femtosecond laser filaments in the nonlinear regime effectively suppresses turbulence-induced beam wander, compared to that in linear propagation. To investigate the effect of varying turbulence strengths, we fixed the phase screen at 2 m and examined the beam wander for both linear and nonlinear propagation. Figure 3 presents the resultant two-dimensional spatial distributions of the beam centroid at the 10 m observation plane under different turbulence refractive index structure constants. For each condition, a statistical analysis was conducted on the centroid positions obtained from 30 independent realizations of random phase screens.

Figure 3a–d show that the mean centroid displacement of the beam spot, ⟨δr⟩, gradually increases with increasing refractive index structure constant $C_{n}^{2}$. For linear propagation, at $C_{n}^{2}$ values of $9.3\times 10^{-14}$ cm^−2/3^, $4.6\times 10^{-13}$ cm^−2/3^, $5.6\times 10^{-13}$ cm^−2/3^, and $1.0\times 10^{-12}$ cm^−2/3^, the corresponding ⟨δr⟩ values are 9.84 μm, 20.69 μm, 22.38 μm, and 31.03 μm, respectively. For nonlinear propagation, at the same set of $C_{n}^{2}$ values, ⟨δr⟩ values are 4.33 μm, 9.20 μm, 10.51 μm, and 14.11 μm, respectively. Furthermore, the two-dimensional spatial distribution of the beam centroid coordinates in Figure 3a–d reveals that under each $C_{n}^{2}$ condition, the mean offset ⟨δr⟩ is consistently smaller for nonlinear propagation than for linear propagation. This indicates that the onset of filamentation under nonlinear conditions effectively suppresses the beam wander induced by turbulence along the propagation path.

**Figure 4:**
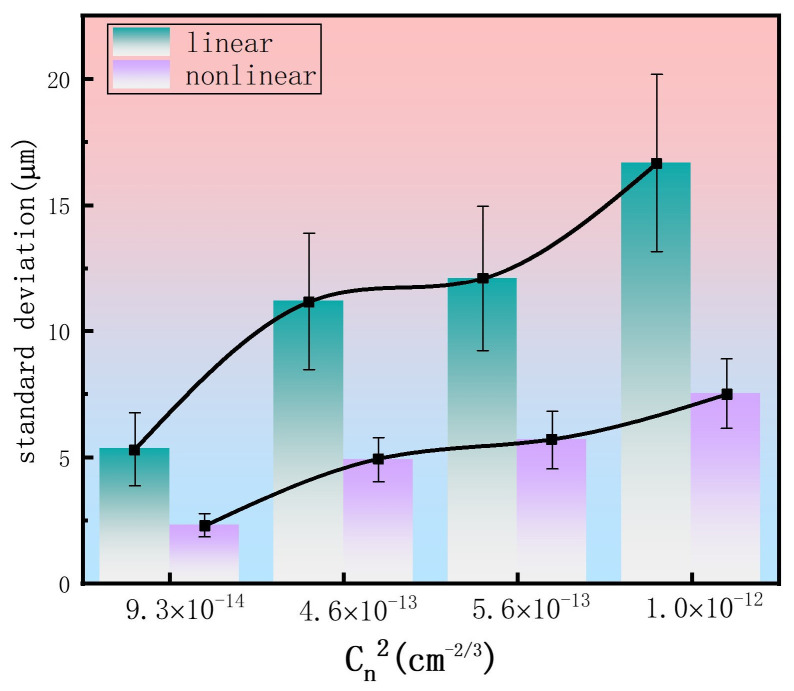
Standard deviation of the centroid displacement for a femtosecond laser beam induced by atmospheric turbulence at a propagation distance of 10 m, plotted as a function of the refractive index structure constant, $C_{n}^{2}$. The black lines connecting the data points are guides to the eye, illustrating the trend of beam wander under different turbulence conditions.

To further analyze the effect of different turbulence intensities on beam wander for both linear and nonlinear propagation, we fixed the turbulence screen at 2 m. We then performed a statistical analysis on the standard deviation of the spot centroid displacement at the 10 m plane under different refractive index structure constants, as shown in Figure 3. The statistical results are summarized in Figure 4.

As shown in Figure 4, an increase in the refractive index structure constant $C_{n}^{2}$ (which characterizes turbulence strength) results in an increased centroid offset of the femtosecond laser spot under both linear and nonlinear propagation conditions. In comparison to the standard deviation of the centroid offset induced by turbulence in linear propagation, the formation of femtosecond laser filaments under nonlinear conditions effectively suppresses this standard deviation, thereby reducing beam wander caused by turbulence along the propagation path. For $C_{n}^{2}$ values of $9.3\times 10^{-14}$ cm^−2/3^, $4.6\times 10^{-13}$ cm^−2/3^, $5.6\times 10^{-13}$ cm^−2/3^, and $1.0\times 10^{-12}$ cm^−2/3^, the standard deviation of the spot centroid offset induced by turbulence at 10-m focal plane under nonlinear conditions is reduced to 43.3%, 43.9%, 47.0%, and 45.1%, respectively, of that under linear conditions. These results demonstrate that, compared to linear propagation, filament formation during nonlinear propagation markedly suppresses turbulence-induced beam wander, leading to a significantly reduced centroid offset. The change in refractive index induced by the Kerr self-focusing effect within a filament is $∆n=n_{2}I$, where *n*_2_ is the nonlinear refractive index coefficient and *I* is the laser intensity. This yields an estimated $∆n\approx 10^{-5}$ (dimensionless). On the other hand, the refractive index fluctuations corresponding to $C_{n}^{2}$ are described by $\langle \bar{{\left[n\left(x+r\right)-n(x)\right]}^{2}}\rangle =C_{n}^{2}r^{2/3}$. Over a unit propagation distance Δz, the spatial refractive index fluctuation induced by atmospheric turbulence can be expressed as $\tilde{n}(x,y,z_{0})=\varphi (x,y,z_{0})/(k_{0}∆z)$, where $k_{0}$ is the wavenumber and $\varphi (x,y,z_{0})$ is the random phase fluctuation imposed by a turbulence screen. Based on this, the refractive index fluctuations $\tilde{n}(x,y,z_{0})$ under our simulation conditions are calculated to be on the order of 10^−6^, which is smaller than the ∆n produced by Kerr self-focusing. Hence, during nonlinear propagation, the refractive-index variation from Kerr self-focusing dominates over that from atmospheric turbulence. This dominance is the underlying mechanism that suppresses turbulence-induced beam wander.

While this work relies on numerical simulations, our key findings are qualitatively consistent with recent experimental observations. Specifically, our simulations show a significant suppression of beam wander via nonlinear self-guiding, confirming the robustness of filamentation in turbulence. This is consistent with the experimental results by [35], which observed a drastic reduction in centroid drift for femtosecond filaments compared to linear propagation. Furthermore, the enhanced structural stability of the vortex beam’s filamentation reported here is corroborated by the experimental study of [24], which demonstrated that vortex pulses maintain superior pointing stability and structural integrity in turbulent air compared to Gaussian beams. These agreements with independent experimental data reinforce the validity of our simulation model.

### 3.2. Comparison Between the Dynamic Evolution of Femtosecond Laser Filamentation in Atmospheric Turbulence for Vortex Beams with Topological Charge l = 2 and Gaussian Beams

In atmospheric turbulence, the propagation quality of optical beams is significantly degraded by turbulence-induced refractive index inhomogeneities. While the preceding section demonstrated that the nonlinear self-guiding effect inherent to filamentation can suppress turbulence-induced beam wander, the influence of the beam’s initial spatial structure on its resilience to turbulence warrants further investigation. This section therefore compares the dynamic evolution of femtosecond laser filamentation in atmospheric turbulence between conventional Gaussian beams and vortex beams carrying orbital angular momentum (OAM), with the latter characterized by a distinctive annular intensity profile with a central phase singularity. This study aims to compare the robustness of a vortex beam (VB) with topological charge l=2 to that of a conventional Gaussian beam (GB) under different levels of turbulence intensity. The input peak power is fixed at $P_{in}=10P_{cr}$, where $P_{cr}$ is the critical power for self-focusing. This allows for a direct comparison of turbulence resistance across different spatial modes. The choice of topological charge l=2 offers an optimal balance, generating a robust four-filament array. This selection aligns with the ongoing exploration of structured beams for turbulence resistance [24].

**Figure 5:**
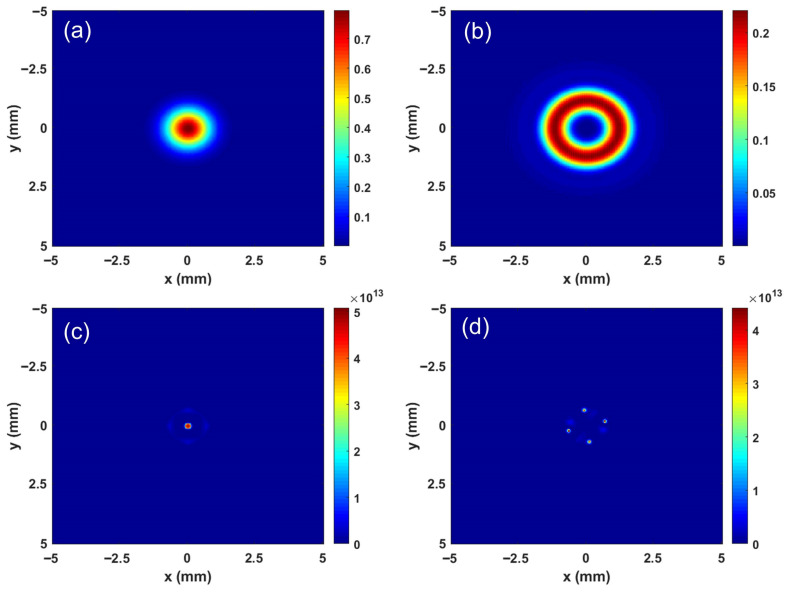
Two-dimensional transverse intensity profiles of the laser beams at propagation distance *z* = 2 m. (**a**) Gaussian beam under linear propagation. (**b**) Vortex beam with a topological charge of *l* = 2 under linear propagation. (**c**) Gaussian beam under nonlinear propagation. (**d**) Vortex beam with a topological charge of *l* = 2 under nonlinear propagation.

The two-dimensional spatial profiles of the optical spots at a propagation distance of 2 m under linear and nonlinear conditions were simulated and are shown in Figure 5. A comparison of these four cases clearly reveals the significant influence of both the beam type (Gaussian or vortex) and the medium’s propagation regime (linear or nonlinear) on the resultant field distribution. Figure 5a shows the case for a Gaussian beam under linear propagation. The standard intensity distribution of a Gaussian beam follows $I\left(r\right)\propto \exp\left(-2r^{2}/w_{0}^{2}\right)$. The spot exhibits a typical circularly symmetric profile, characterized by a bright central peak where the intensity is highest, followed by a rapid decay toward the gradually dimmer edges. This is the expected outcome for a Gaussian beam propagating in a linear medium (i.e., in the absence of nonlinearities), where it maintains its fundamental shape with only minor diffraction-induced broadening. Figure 5b presents the results for a vortex beam with topological charge l=2 under linear propagation. Such beams carry orbital angular momentum, characterized by a helical phase front which is described by $\exp\left(il\varphi \right)$. The resulting beam profile exhibits a characteristic ring structure, featuring a central dark core with near-zero intensity surrounded by a bright annular region. This ring pattern arises directly from the helical phase structure, which creates a phase singularity at the beam center. This singularity, known as an optical vortex, manifests as the observed null intensity (dark core). The topological charge l=2 indicates that the phase completes two full 2π rotations around the axis, which typically stabilizes the ring-like intensity profile. Figure 5c shows the scenario for a Gaussian beam under nonlinear propagation. In this nonlinear regime, the Gaussian beam experiences Kerr self-focusing. This effect counteracts diffraction and compresses the beam, leading to extreme energy concentration at the center and the formation of an intense, localized filament—resulting in a much smaller and brighter spot. Finally, Figure 5d presents the case of a vortex beam (l=2) under nonlinear propagation. Here, the central dark core vanishes and is replaced by several discrete, symmetrically arranged bright spots. This is the multifilamentation phenomenon observed when vortex beams propagate in a nonlinear medium. This multifilamentation effect occurs because the nonlinearity disrupts the integrity of the initial optical phase singularity. Consequently, the initially continuous annular intensity profile breaks up into multiple stable subfilaments.

**Figure 6:**
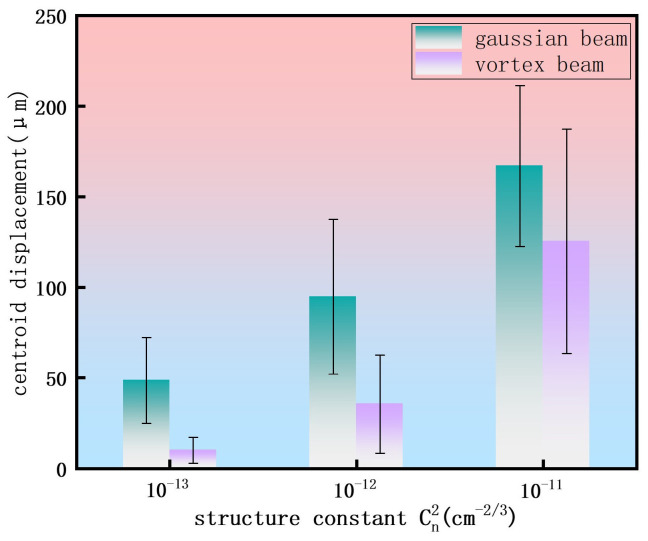
Centroid displacements and their standard deviations for 30 independent realizations of femtosecond laser filamentation at a propagation distance of 2 m. The results compare a Gaussian beam and a vortex beam with a topological charge l=2 propagating through atmospheric turbulence with refractive index structure constants of $10^{-13}$ cm^−2/3^, $10^{-12}$ cm^−2/3^, and $10^{-11}$ cm^−2/3^.

We employed numerical simulations to statistically analyze the propagation characteristics of two beam types—vortex and Gaussian beams—under three representative atmospheric refractive index structure constants at a propagation distance of 2 m. Figure 6 shows the centroid displacement and its standard deviation for both beams at 2 m under different turbulence conditions. The data reveal an increasing trend in centroid displacement with escalating turbulence strength for both beams; however, the vortex beam consistently demonstrates superior stability. Under weak turbulence conditions ($C_{n}^{2}=10^{-13}$ cm^−2/3^), the centroid displacement of a vortex beam with topological charge l=2 is only 20.66% of that of Gaussian beams. Even under strong turbulence conditions ($C_{n}^{2}=10^{-11}$ cm^−2/3^), the l=2 vortex beam maintains a beam centroid displacement of approximately 125.52 μm, which is lower than the 167.02 μm observed for the Gaussian beam. All data points are derived from 30 independent random phase screen simulations, with error bars representing the standard deviation. This research confirms that a vortex beam with topological charge l=2 exhibits enhanced propagation stability in atmospheric turbulence compared to a Gaussian beam. This advantage persists with increasing turbulence intensity, providing new insights into the physical mechanisms relevant to long-range free-space optical communication and laser atmospheric sensing applications.

**Figure 7:**
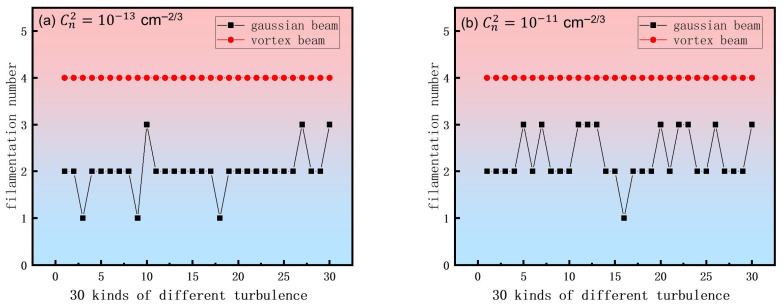
Statistical analysis of the number of femtosecond laser filaments at a propagation distance of 2 m for different types of incident beams (Gaussian and vortex). (**a**) Refractive index structure constant $C_{n}^{2}=10^{-13}$ cm^−2/3^. (**b**) Refractive index structure constant $C_{n}^{2}=10^{-11}$ cm^−2/3^.

As shown in Figure 7, to elucidate the superior resistance to atmospheric turbulence of vortex beams over Gaussian beams, we quantified the number of filaments generated by Gaussian and vortex beams at a propagation distance of 2 m for different turbulence intensities. The statistical results, presented in Figure 7, were obtained from simulations using 30 independent random phase screens. Figure 7a corresponds to a weak turbulence regime, with a refractive index structure constant of $C_{n}^{2}=10^{-13}$ cm^−2/3^. Under these conditions, the vortex beam consistently produced four filaments in all 30 simulations, whereas the filament count for the Gaussian beam varied stochastically between one and three. Figure 7b represents a strong turbulence regime ($C_{n}^{2}=10^{-11}$ cm^−2/3^). Notably, the vortex beam maintained a constant filament number of four, while the Gaussian beam’s count continued to vary within the same range but displayed greater fluctuations. To further explore the limits of the proposed strategy, we extended simulations to ultra-strong turbulence regimes with $C_{n}^{2}$ up to ${5\times 10}^{-11}$ cm^−2/3^. Results show that while the nonlinear self-guiding effect remains dominant for $C_{n}^{2}<{3\times 10}^{-11}$ cm^−2/3^, its relative suppression efficiency diminishes slightly beyond this threshold due to the increased magnitude of turbulent phase distortions approaching the nonlinear phase shift. Nevertheless, even at $C_{n}^{2}={5\times 10}^{-11}$ cm^−2/3^, the vortex beam configuration maintained a stable four-filament array, whereas the Gaussian beam exhibited stochastic fragmentation. This suggests that the practical application limit for robust operation in open atmosphere is approximately $C_{n}^{2}\approx {3\times 10}^{-11}$ cm^−2/3^, beyond which adaptive correction might be required in conjunction with the proposed passive strategies.

In summary, the results highlight a fundamental difference: the Gaussian beam is highly sensitive to turbulence, leading to an unstable filamentation process. In contrast, the vortex beam exhibits remarkable robustness, with its filament number remaining constant regardless of the turbulence strength.

The consistent generation of four filaments for the l=2 vortex beam, in contrast to the stochastic filamentation of Gaussian beams, can be attributed to azimuthal modulation instability (AMI) inherent to high-power vortex beams. Theoretical analyses indicate that the number of filaments emerging from a vortex beam is determined by the topological charge l and the input power, often following a symmetry-breaking pattern where the annular ring splits into N sub-filaments [25,26]. In our configuration, the parameters favor a breakup into four distinct lobes. Crucially, the conservation of orbital angular momentum (OAM) and the central phase singularity impose a structured constraint on this breakup process. Unlike Gaussian beams, where turbulence randomly seeds multiple filaments, the OAM-carrying structure resists random perturbations, forcing the energy to concentrate into the deterministic modes dictated by the beam’s helical phase. This intrinsic structural stability ensures that the filament number remains fixed at four, even under varying turbulence strengths, as the nonlinear dynamics driven by the phase singularity dominate over turbulence-induced noise.

Gaussian beams in the TEM_00_ mode are characterized by a strongly peaked central intensity profile. When subjected to atmospheric turbulence, this central lobe is highly susceptible to severe attenuation or scattering, resulting in uncontrolled variation in the number of filaments generated. In contrast, vortex beams carry orbital angular momentum (OAM) and feature a distinctive annular intensity distribution with a central phase singularity. This unique structure confers upon vortex beams enhanced resilience against aberration-induced distortion during propagation through turbulent atmosphere. Consequently, they maintain a stable filament count and preserve the requisite energy threshold for filamentation even in complex atmospheric environments. Our numerical findings demonstrate that for long-range, high-reliability atmospheric laser transmission applications—such as LIDAR and atmospheric monitoring—vortex beams outperform conventional Gaussian beams, offering more stable detection signals under adverse weather or strong turbulence conditions.

Practically, high-power femtosecond vortex beams can be realized by integrating spiral phase plates into high-energy laser amplifiers, a method proven to sustain high damage thresholds [36]. Although atmospheric turbulence induces OAM mode degradation and crosstalk [37], our results suggest that the nonlinear filamentation regime mitigates severe performance loss by maintaining a stable multi-filament energy distribution. Future engineering implementations may further employ adaptive optics to pre-correct wavefront distortions, ensuring robust operation in complex environments.

## 4. Conclusions

This study systematically investigated the impact of atmospheric turbulence on the centroid wander of femtosecond laser beams through systematic numerical simulations and elucidated the underlying suppression mechanism inherent to the nonlinear filamentation process. The results demonstrated that filament formation can significantly suppress beam wander induced by atmospheric turbulence. Simulations reveal that for refractive index structure constants $C_{n}^{2}$ of $4.6\times 10^{-13}$ cm^−2/3^, $5.6\times 10^{-13}$ cm^−2/3^, and $1.0\times 10^{-12}$ cm^−2/3^, the standard deviation of the beam centroid displacement at the 10-m focal plane under nonlinear conditions is reduced to 43.9%, 47.0%, and 45.1%, respectively, of that observed under linear propagation. Theoretical analysis indicates that this physical mechanism originates from the change in refractive index induced by the Kerr effect within the filament ($∆n\approx 10^{-5}$, dimensionless), which is an order of magnitude larger than the atmospheric turbulence-induced refractive index fluctuation ($\tilde{n}\approx 10^{-6}$, dimensionless), thereby dominating the beam propagation dynamics. Furthermore, the study shows that the disruptive effect of turbulence weakens as the turbulent layer approaches the focal region. Additional investigations demonstrate that vortex beams carrying orbital angular momentum can not only effectively mitigate atmospheric turbulence-induced distortions through nonlinear effects but also inhibit the stochastic generation of multiple filaments, thereby enhancing the stability of filamentary propagation. The research results can be integrated into the beam modulation and emission modules of laser sensors and LIDAR systems for atmospheric pollutant remote sensing, which significantly improves the anti-turbulence performance of such optical sensors and enhances their detection stability and effective working range in open atmospheric environments. This work provides profound theoretical insights and suggests viable strategies for suppressing turbulent effects in the atmospheric transmission of femtosecond lasers, thereby enabling promising applications in fields such as free-space optical communication and laser remote sensing.

## Data Availability

The data presented in this study are available upon request from the corresponding author.
